# Effects of Different Maturity Groups on Photosynthetic Performance, Grain-Filling Dynamics, and Yield of Maize Under Film Mulching in a Cool Region

**DOI:** 10.3390/plants15172596

**Published:** 2026-08-25

**Authors:** Yangming Guo, Hanglin Song, Danhui Zhang, Chen Xu, Fei Li, Lihua Zhang, Bo Qu, Shaofeng Bian, Hongxiang Zhao, Ning Sun

**Affiliations:** 1 Faculty of Agronomy, Jilin Agricultural University, Changchun 130118, China; guoyangming1996@mails.jlau.edu.cn (Y.G.);; 2 Institute of Agricultural Resources and Environment, Jilin Academy of Agriculture Sciences, Changchun 130033, China; songhanglin@jaas.com.cn (H.S.);; 3 Multidisciplinary, Observation and Research Station, Ministry of Agriculture and Rural Affairs, Taonan 137100, China

**Keywords:** biodegradable film mulching, maize, maturity group, photosynthetic traits, grain filling, starch synthesis, grain yield

## Abstract

Spring maize production in cool regions of Northeast China is constrained by low temperatures, variable soil moisture, and limited thermal resources. A two-year field experiment was conducted in Dunhua, Jilin Province, China, in 2024 and 2025 to evaluate the growth, physiological performance, grain-filling characteristics, starch accumulation, and yield of six maize hybrids representing early-, medium–early-, and medium-maturing groups under biodegradable film mulching. Soil temperature and moisture content, photosynthetic traits, shoot dry matter accumulation, grain-filling parameters, starch synthase activity, starch content, and grain yield were measured. The medium–early-maturing group maintained higher net photosynthetic rate, stomatal conductance, and transpiration rate at the twelve-leaf stage (V12) and milk stage (R3) and accumulated more shoot dry matter at R3. Compared with the early-maturing group, the maximum grain-filling rate (Rmax), kernel weight at the maximum grain-filling rate (Wmax), and mean grain-filling rate (Vmean) increased by 10.0%, 12.1%, and 7.2%, respectively; compared with the medium-maturing group, the corresponding increases were 5.4%, 2.8%, and 9.0%, respectively. The medium–early-maturing group also maintained relatively high starch synthase activity and starch accumulation during the middle and late grain-filling stages. Averaged across the two years, grain yield was 20.0% and 9.1% higher than that of the early- and medium-maturing groups, respectively. Overall, the medium–early-maturing group achieved better coordination among canopy photosynthesis, dry matter accumulation, grain filling, and starch synthesis and showed better adaptation to the thermal conditions of this agroecological region, where the annual effective accumulated temperature is approximately 2300–2400 °C·d. Therefore, the medium–early-maturing group may represent a suitable option for achieving high yield under biodegradable film mulching in this region.

## 1. Introduction

Northeast China is one of the country’s most important production regions for spring maize (*Zea mays* L.). However, maize production in its cool regions is constrained by slow soil warming in spring, a short frost-free period, and limited thermal resources. These conditions can delay maize emergence, restrict early vegetative growth, and impair crop stand establishment, ultimately reducing photosynthate accumulation and grain yield formation [1]. Under such environmental conditions, film mulching can improve the soil hydrothermal environment during the early growing season by increasing topsoil temperature and reducing soil water evaporation. These effects promote maize emergence and early growth, enhance leaf area expansion and canopy photosynthetic capacity, and provide a physiological basis for subsequent dry matter accumulation, assimilate translocation, and grain filling [2,3,4,5,6]. With ongoing changes in regional thermal conditions and adjustments in the maturity-group composition of maize hybrids, coordinating maturity groups, sowing date, and regional thermal resources has become an important strategy for improving maize production potential in cool regions [7].

The maturity of a maize hybrid reflects the thermal requirement needed to complete its developmental cycle, and its regional adaptability largely depends on the degree of correspondence between this thermal requirement and the locally available accumulated temperature [8,9,10]. Maize hybrids suitable for cultivation in Northeast China can be classified into different maturity groups according to the length of their growing periods. In production practice, the accumulated temperature required by a hybrid is generally expected to be lower than the long-term accumulated temperature available in the target region to ensure physiological maturity before the onset of autumn chilling or frost [11,12]. Within this context, moderately extending the crop growth period may increase the duration of post-silking photosynthesis and dry matter accumulation. However, whether this longer growth period can be translated into a yield advantage depends on the match between the thermal requirement of the hybrid and the available regional thermal resources, particularly during the post-silking period [13,14,15]. Differences in thermal requirements and developmental rhythms among maize maturity groups not only determine crop phenology [9] but also influence canopy establishment, photosynthetic production, assimilate partitioning, and kernel development [16,17]. Hybrids with lower thermal requirements can enter the grain-filling and maturity stages earlier, which helps them avoid the risks of low temperatures during the late growing season and restricted kernel dehydration. However, their shorter leaf area duration and photosynthetically active period may limit canopy dry matter accumulation and kernel sink capacity [18]. Hybrids with higher thermal requirements generally have a larger leaf area, a longer photosynthetically active period, and greater biomass production potential [19]. Nevertheless, the realization of their yield advantage depends on sufficient post-silking thermal resources. In cool regions, insufficient accumulated temperature during the late growing season may lead to a decline in photosynthetic activity, a delay in the peak grain-filling period, restricted kernel dehydration, and limited starch accumulation [14,20]. By contrast, hybrids with moderate thermal requirements may achieve a more favorable balance between maturity security and post-silking productivity, thereby maintaining closer temporal coordination among leaf area expansion, photosynthetic carbon assimilation, dry matter accumulation, and kernel sink demand [21,22,23]. Therefore, although film mulching improves the hydrothermal conditions for early maize growth, the extent to which different maturity groups convert this advantage into enhanced post-silking photosynthesis, grain-filling efficiency, and yield may depend on how their developmental processes respond to regional thermal conditions [22,23,24].

Biodegradable mulch film can retain the warming and soil moisture conservation functions of conventional film mulching while reducing the environmental risks associated with residual polyethylene film [25]. However, previous studies have primarily focused on the physical properties of mulch materials or the growth responses of a single maize hybrid. The regional adaptability of maize hybrids from contrasting maturity groups under biodegradable film mulching remains poorly understood. In particular, limited information is available on how differences among maturity groups affect yield formation through changes in canopy growth, leaf photosynthesis, dry matter accumulation, grain filling, and starch synthesis [25,26,27]. Early-maturing maize hybrids are currently predominant in local production systems. Therefore, in the present study, the locally cultivated early-maturing group was used as a production reference and compared with medium–early- and medium-maturing groups under identical biodegradable film mulching and crop management conditions. Soil temperature and moisture content, crop growth, leaf photosynthetic characteristics, dry matter accumulation, grain-filling characteristics, starch synthesis, and grain yield were systematically evaluated among the different maturity groups. The objectives of this study were to: (1) characterize the physiological responses and yield formation processes of maize hybrids from contrasting maturity groups under biodegradable film mulching; (2) evaluate the productivity and adaptation to local thermal conditions of medium–early- and medium-maturing groups relative to the locally cultivated early-maturing group; and (3) identify a suitable maize maturity group that balances maturity security with yield potential in the cool regions of Northeast China. We hypothesized that the medium–early-maturing group would show better adaptation to local thermal conditions, thereby coordinating canopy photosynthesis, dry matter production, starch synthesis, and kernel filling to produce a greater grain yield under biodegradable film-mulched cultivation.

## 2. Results

### 2.1. Effects of Mulch Cover on Soil Temperature and Moisture Content

**Figure 1:**
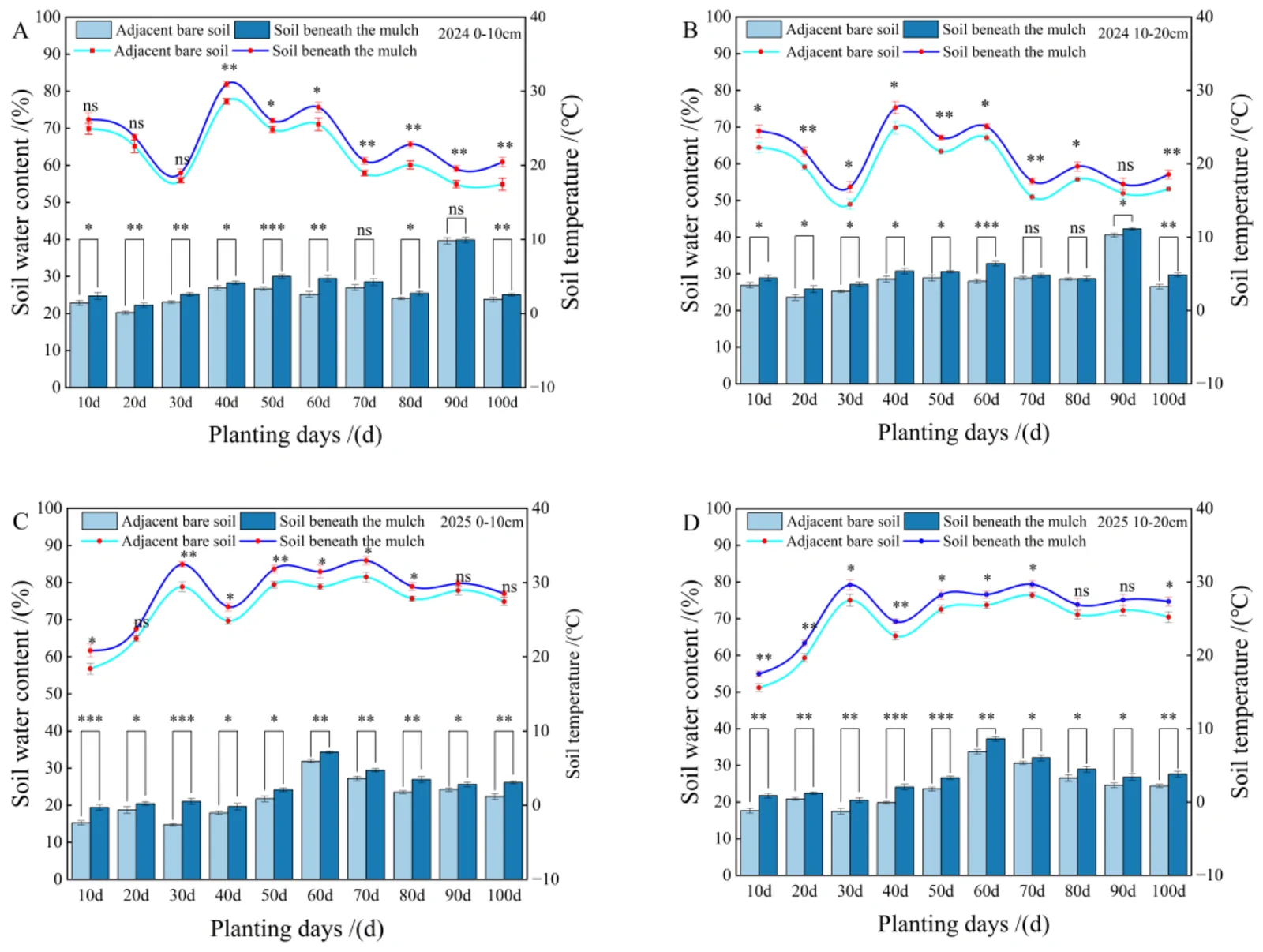
Temporal dynamics of soil moisture content and soil temperature at different soil depths beneath the mulch and at the uncovered position within each plot during the 2024 and 2025 maize growing seasons. (**A**) The 0–10 cm soil layer in 2024; (**B**) the 10–20 cm soil layer in 2024; (**C**) the 0–10 cm soil layer in 2025; and (**D**) the 10–20 cm soil layer in 2025. Note: Bars represent soil moisture content, and lines represent soil temperature. *, **, and *** indicate significant differences between the uncovered position and the position beneath the mulch at *p* < 0.05, *p* < 0.01, and *p* < 0.001, respectively; ns indicates no significant difference. The comparison represents a within-plot positional comparison between the position beneath the mulch and the uncovered position rather than a randomized comparison between mulched and unmulched treatments.

As shown in Figure 1, soil temperature and moisture content in the 0–10 and 10–20 cm soil layers were generally higher beneath the mulch than at the uncovered position within each plot in both 2024 and 2025. During the early and middle growth stages of maize, these positional differences were more pronounced in both soil layers. As maize growth progressed and the biodegradable mulch film gradually degraded, the differences between the two positions became smaller during the late growth stages. Throughout the observation period, significant differences in soil temperature and moisture content between the two positions were observed at multiple sampling dates and soil depths (*p* < 0.05). In 2024, compared with the uncovered position, soil moisture content beneath the mulch was higher by 0.7–17.3% and 0.4–17.2% in the 0–10 and 10–20 cm soil layers, respectively, while soil temperature was higher by 4.80–17.19% and 6.5–16.2%, respectively. Similar differences were observed in 2025, when soil moisture content beneath the mulch was higher by 5.8–42.6% and 4.6–23.3% in the 0–10 and 10–20 cm soil layers, respectively, and soil temperature was higher by 3.3–13.3% and 5.3–12.1%, respectively.

### 2.2. Photosynthetic Traits of Maize Hybrids from Different Maturity Groups Under Film Mulching

**Figure 2:**
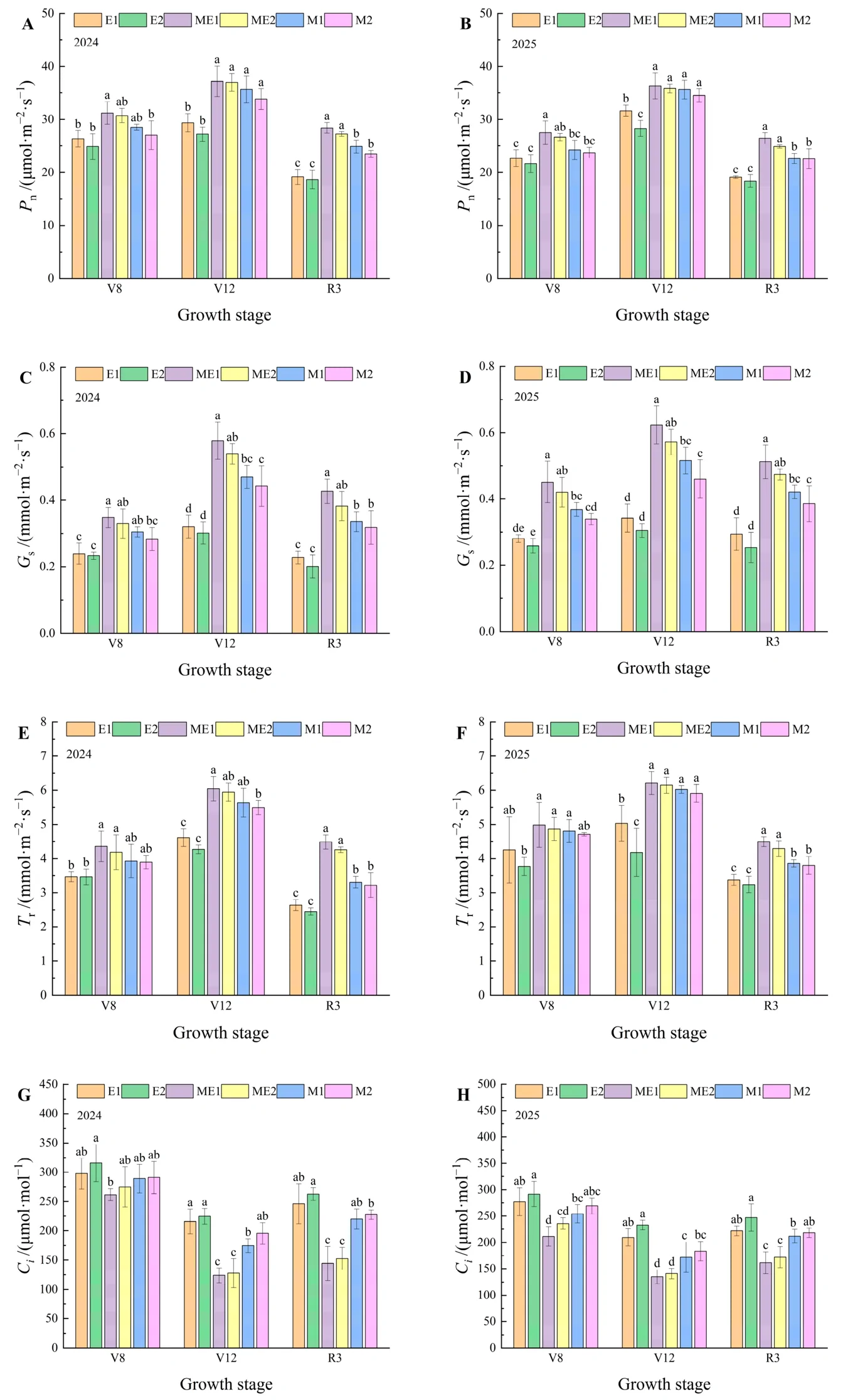
Leaf photosynthetic gas-exchange traits of maize hybrids from different maturity groups under biodegradable film mulching at the eight-leaf stage (V8), twelve-leaf stage (V12), and milk stage (R3) in 2024 and 2025. (**A**) Net photosynthetic rate (*P*_n_) in 2024; (**B**) net photosynthetic rate (*P*_n_) in 2025; (**C**) stomatal conductance (*G*_s_) in 2024; (**D**) stomatal conductance (*G*_s_) in 2025; (**E**) transpiration rate (*T*_r_) in 2024; (**F**) transpiration rate (*T*_r_) in 2025; (**G**) intercellular CO_2_ concentration (*C*_i_) in 2024; and (**H**) intercellular CO_2_ concentration (*C*_i_) in 2025. Note: Different lowercase letters indicate significant differences among maize hybrids within the same year and growth stage at *p* < 0.05.

Changes in the photosynthetic traits of maize hybrids from different maturity groups under film mulching are shown in Figure 2. At V12 and R3 in both 2024 and 2025, the maturity-group means of net photosynthetic rate (Pn), stomatal conductance (Gs), and transpiration rate (Tr) generally followed the order medium–early-maturing > medium-maturing > early-maturing. Averaged across the two years, compared with the early-maturing group, Pn increased by 40.4% and 21.6%, Gs by 82.4% and 50.0%, and Tr by 40.2% and 24.9% in the medium–early- and medium-maturing groups, respectively. In contrast, the maturity-group mean intercellular CO_2_ concentration (Ci) at V12 and R3 generally followed the order early-maturing > medium-maturing > medium–early-maturing. Averaged across the two years, compared with the medium–early-maturing group, Ci was 60.8% and 38.5% higher in the early- and medium-maturing groups, respectively.

As shown in Table 1, year, maturity group, and growth stage had significant main effects on *P*n, *G*s, *T*r, and *C*i (*p* < 0.01). The interaction between year and maturity group had no significant effect on any of the four photosynthetic parameters. The interaction between year and growth stage had a highly significant effect on *P*n (*p* < 0.001), a significant effect on *T*r (*p* < 0.05), and a highly significant effect on *C*i (*p* < 0.01), but had no significant effect on *G*s. The interaction between maturity group and growth stage had highly significant effects on *P*n, *G*s, *T*r, and *C*i (*p* < 0.01). The three-way interaction among year, maturity group, and growth stage had no significant effect on any of the four photosynthetic parameters.

### 2.3. Effects of Maturity Group on the Grain-Filling Characteristics of Maize Under Film Mulching

**Figure 3:**
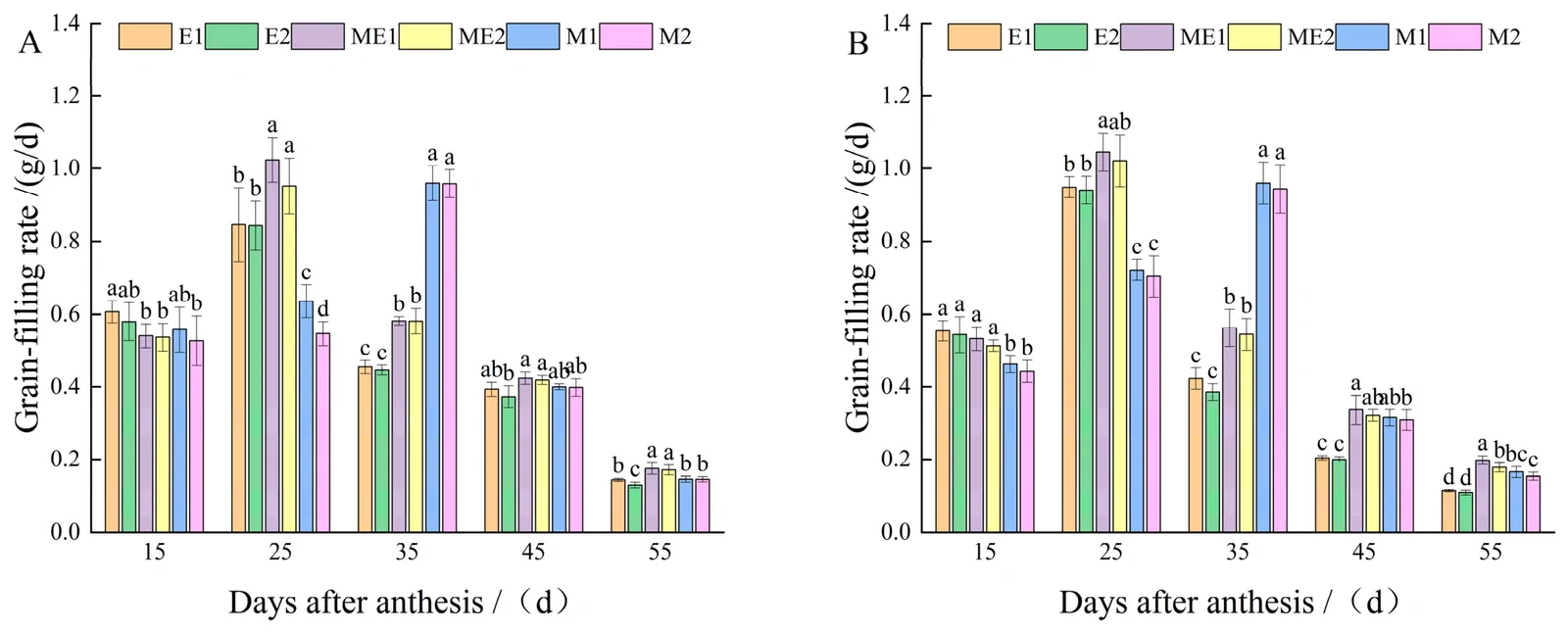
Grain-filling rates of maize hybrids from different maturity groups on different days after anthesis under biodegradable film mulching in 2024 and 2025. (**A**) Grain-filling rate in 2024; (**B**) grain-filling rate in 2025. Note: Different lowercase letters above the bars indicate significant differences among maturity groups on the same day after anthesis within the same year at *p* < 0.05.

Figure 3 shows the changes in grain-filling rate during the first 60 days after anthesis in 2024 and 2025 under film mulching. The grain-filling rates of all maturity groups exhibited a parabolic pattern, increasing initially and then decreasing. During the first 30 days after anthesis, the early- and medium–early-maturing groups maintained relatively high grain-filling rates. At 25 days after anthesis, the grain-filling rates of the early- and medium–early-maturing groups were significantly higher than those of the medium-maturing group (*p* < 0.05), with average increases of 37.2% and 55.0%, respectively, across the two years. The grain-filling rate of the medium-maturing group peaked at 35 days after anthesis and was significantly higher than that of the early- and medium–early-maturing groups (*p* < 0.05), with average increases of 123.6% and 68.4%, respectively, across the two years.

As shown in Table 2, grain-filling parameters differed among maturity groups under film mulching. Across the two years, the maximum grain-filling rate (Rmax), kernel weight at the maximum grain-filling rate (Wmax), and mean grain-filling rate (Vmean) of the medium–early-maturing group were generally higher than those of the early- and medium-maturing groups. Compared with the early- and medium-maturing groups, the medium–early-maturing group showed average increases of 10.0% and 5.4% in Rmax, 12.1% and 2.8% in Wmax, and 7.2% and 9.0% in Vmean, respectively, across the two years. In contrast, the time to the maximum grain-filling rate (Tmax) and effective grain-filling duration (t3) of the medium-maturing group were significantly greater than those of the early- and medium–early-maturing groups (*p* < 0.05). Compared with the early- and medium–early-maturing groups, Tmax was prolonged by 24.7% and 13.6%, respectively, whereas t3 was prolonged by 11.7% and 6.4%, respectively. Two-way ANOVA showed that year had significant effects on Rmax, Tmax, Vmean, and t3 (*p* < 0.01), but had no significant effect on Wmax. Maturity group had significant effects on all grain-filling parameters, including Rmax, Tmax, Wmax, Vmean, and t3 (*p* < 0.01). The interaction between year and maturity group significantly affected Rmax, Tmax, and t3 (*p* < 0.01), whereas no significant interaction effects were observed for Wmax or Vmean.

Based on the Logistic model, the grain-filling process of each maturity group was further divided into three stages: the early, middle, and late grain-filling stages (Table 3). Kernel weight in all maturity groups exhibited a typical sigmoidal pattern, characterized by slow accumulation during the early stage, rapid accumulation during the middle stage, and a gradual reduction in the increment during the late stage. Both the kernel weight increment and grain-filling rate were greatest during the middle grain-filling stage. Although the late grain-filling stage had the longest duration, it exhibited the lowest grain-filling rate. During the middle grain-filling stage, the grain-filling rate of the medium–early-maturing group was significantly higher than that of the early- and medium-maturing groups, with average increases of 9.9% and 4.7%, respectively, across the two years. Similarly, the kernel weight increment of the medium–early-maturing group during the middle grain-filling stage was significantly higher than that of the early- and medium-maturing groups, with average increases of 12.1% and 2.8%, respectively, across the two years.

### 2.4. Effects of Maturity Group on Starch Synthase Activity and Starch Content in Maize Under Film Mulching

**Figure 4:**
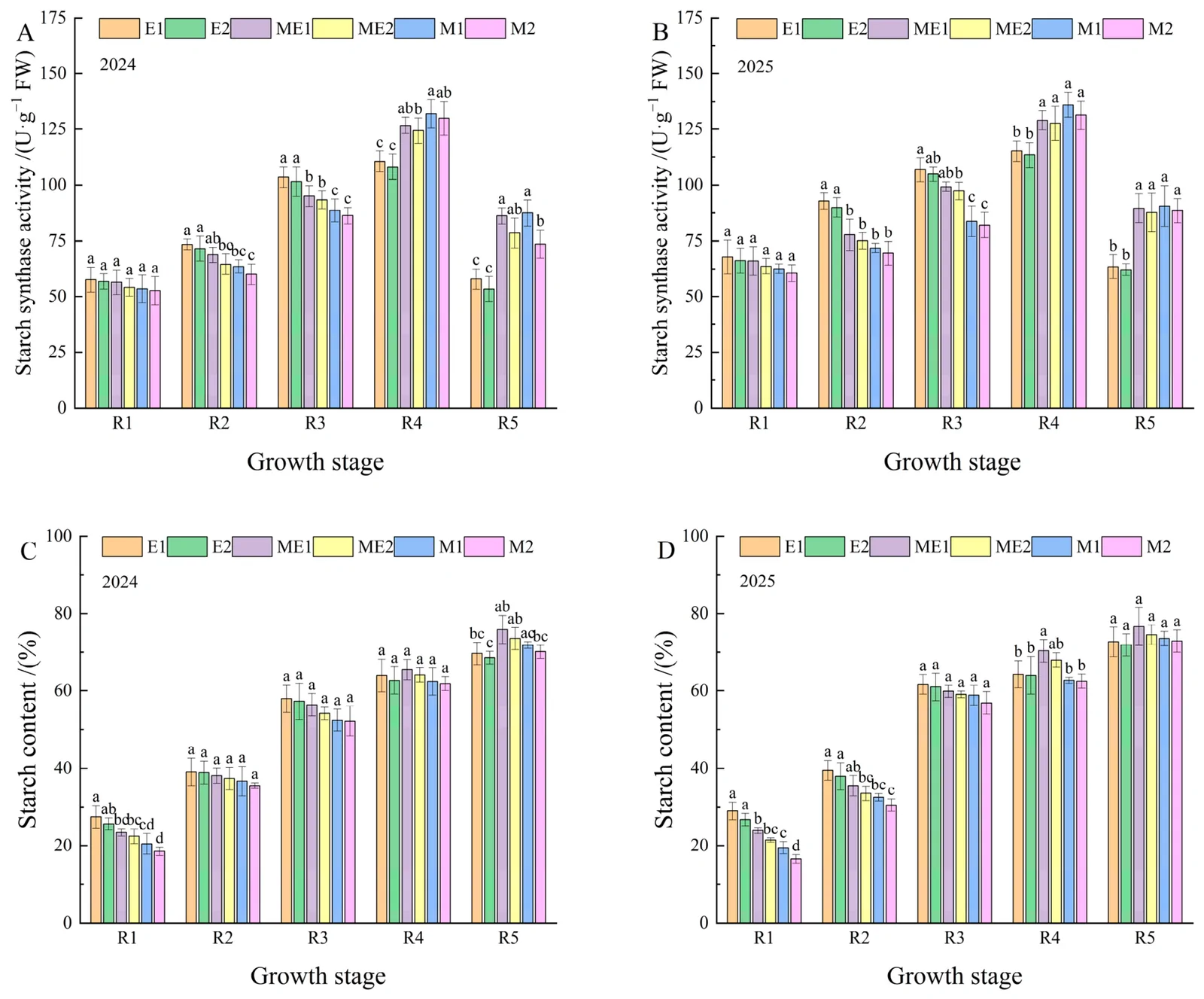
Starch synthase activity and starch content of maize hybrids from different maturity groups at the silking stage (R1), blister stage (R2), milk stage (R3), dough stage (R4), and dent stage (R5) under biodegradable film mulching. (**A**) Starch synthase activity at R1–R5 in 2024; (**B**) starch synthase activity at R1–R5 in 2025; (**C**) starch content at R1–R5 in 2024; and (**D**) starch content at R1–R5 in 2025. Note: Different lowercase letters above the bars indicate significant differences among maturity groups within the same year and growth stage at *p* < 0.05.

Changes in starch synthase activity (SSA) and starch content (S) in maize from different maturity groups from the silking stage (R1) to the dent stage (R5) under film mulching are shown in Figure 4. Across both years, SSA in all maturity groups initially increased and then decreased, reaching its maximum at the dough stage (R4). From R1 to R3, SSA was generally higher in the early-maturing group than in the medium–early- and medium-maturing groups. At R4 and R5, however, SSA in the medium–early- and medium-maturing groups was significantly higher than that in the early-maturing group (*p* < 0.05), with average increases of 24.2% and 27.1%, respectively, across the two years. These results indicate that the medium–early- and medium-maturing groups maintained higher starch synthase activity during the middle and late grain-filling stages. Kernel starch content increased continuously as maize development progressed. The increase was relatively rapid from R1 to R3 but gradually slowed from R3 to R5. From R1 to R3, starch content was generally higher in the early-maturing group than in the medium–early- and medium-maturing groups. As grain filling progressed, the differences among maturity groups gradually decreased. By R5, starch content was generally higher in the medium–early- and medium-maturing groups than in the early-maturing group. Overall, starch accumulation began earlier in the early-maturing group, whereas the medium–early- and medium-maturing groups exhibited a stronger capacity for sustained starch accumulation during the middle and late grain-filling stages.

### 2.5. Effects of Maturity Group on Maize Growth, Development, and Dry Matter Accumulation Under Film Mulching

**Figure 5:**
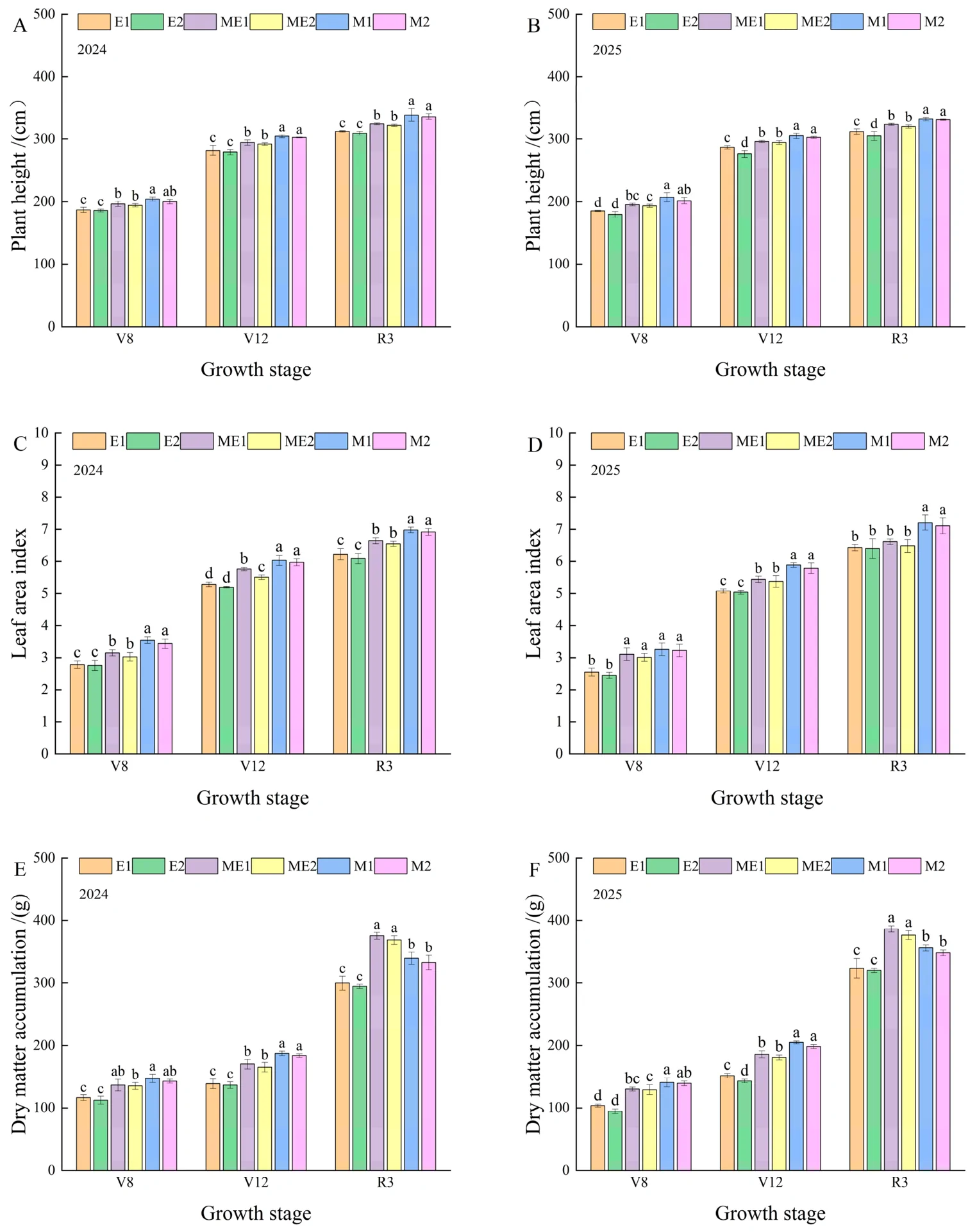
Plant height (PH), leaf area index (LAI), and shoot dry matter accumulation (SDM) of maize hybrids from different maturity groups at the eight-leaf stage (V8), twelve-leaf stage (V12), and milk stage (R3) under biodegradable film mulching. (**A**) Plant height at V8, V12, and R3 in 2024; (**B**) plant height at V8, V12, and R3 in 2025; (**C**) leaf area index at V8, V12, and R3 in 2024; (**D**) leaf area index at V8, V12, and R3 in 2025; (**E**) shoot dry matter accumulation at V8, V12, and R3 in 2024; and (**F**) shoot dry matter accumulation at V8, V12, and R3 in 2025. Note: Different lowercase letters above the bars indicate significant differences among maturity groups within the same year and growth stage at *p* < 0.05.

Plant height (PH), leaf area index (LAI), and shoot dry matter accumulation (SDM) of maize from different maturity groups under film mulching are shown in Figure 5. In both years, PH, LAI, and SDM at V8 and V12 generally followed the order medium-maturing > medium–early-maturing > early-maturing. The values for the medium–early- and medium-maturing groups were significantly higher than those for the early-maturing group (*p* < 0.05). At R3, SDM was significantly higher in the medium–early-maturing group than in the early- and medium-maturing groups, with average increases of 21.7% and 6.4%, respectively, across the two years.

Table 4 presents the effects of year, maturity group, growth stage, and their interactions on plant height (PH), leaf area index (LAI), and shoot dry matter accumulation (SDM). Maturity group and growth stage had significant effects on PH, LAI, and SDM (*p* < 0.01). Year significantly affected LAI and SDM (*p* < 0.01). The interaction between maturity group and growth stage significantly affected SDM (*p* < 0.01), indicating that biomass accumulation among maturity groups varied across growth stages. The three-way interaction among year, maturity group, and growth stage had no significant effect on PH or SDM.

### 2.6. Effects of Maturity Group on Maize Yield and Yield Components Under Film Mulching

As shown in Table 5, the number of grains per ear and grain yield in both 2024 and 2025 generally followed the order of medium–early-maturing > medium-maturing > early-maturing groups. Grain yield was significantly higher in the medium–early-maturing group than in the early- and medium-maturing groups (*p* < 0.05), with average increases of 20.0% and 9.1%, respectively, across the two years. Two-way ANOVA showed that maturity group had highly significant effects on the number of grains per ear, 100-grain weight, and grain yield (*p* < 0.01), whereas year and the interaction between year and maturity group had no significant effects on these variables (*p* > 0.05). These results indicate that yield variation under film mulching was mainly associated with maturity group differences rather than annual variation.

### 2.7. Correlation Analysis

**Figure 6:**
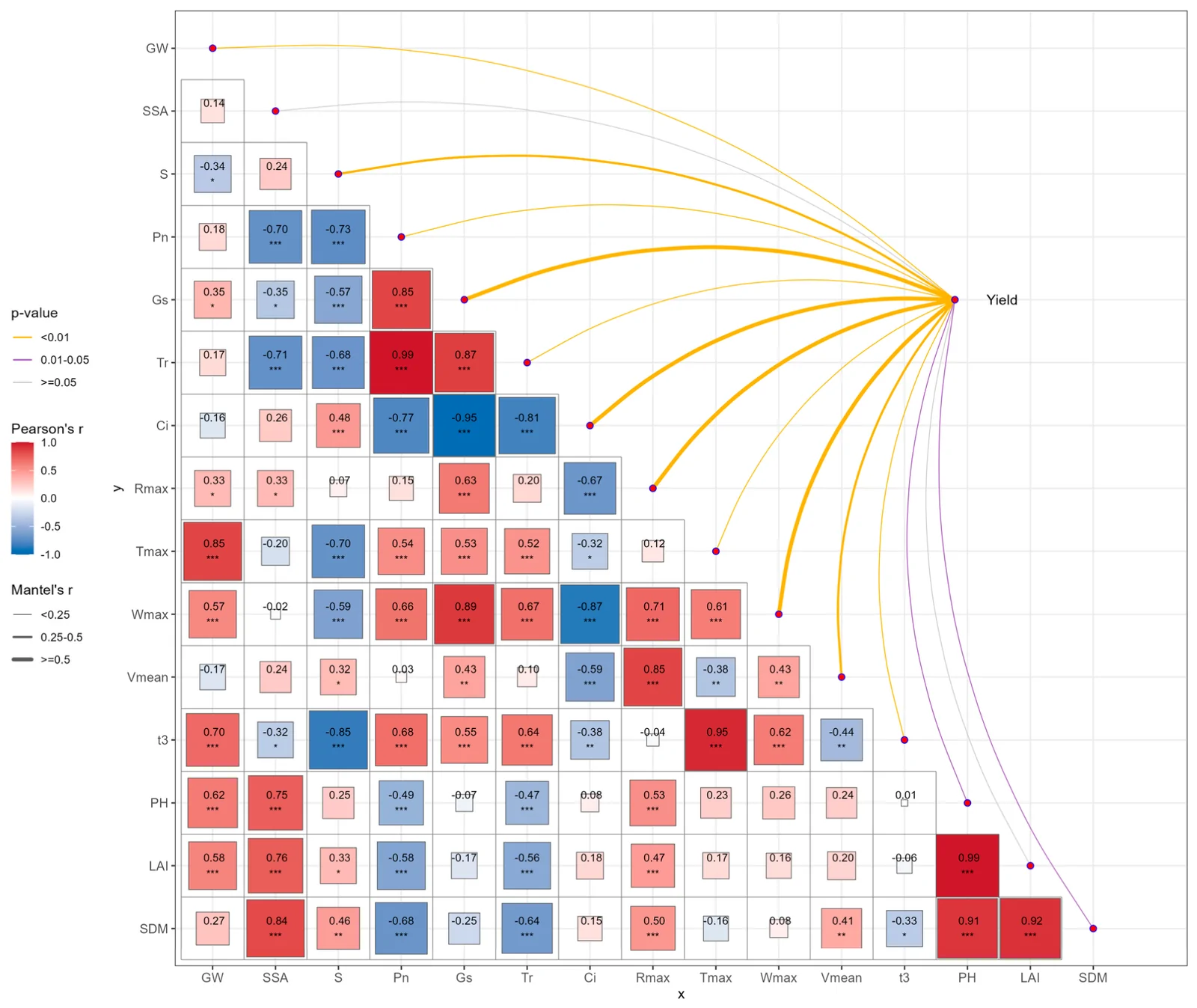
Correlations among maize traits of different maturity groups under film mulching. Note: *, **, and *** indicate significant correlations at *p* < 0.05, *p* < 0.01, and *p* < 0.001, respectively. GW, 100-kernel weight; SSA, starch synthase activity; S, starch content; *P*_n_, net photosynthetic rate; *G*_s_, stomatal conductance; *T*_r_, transpiration rate; *C*_i_, intercellular CO_2_ concentration; R_max_, maximum grain-filling rate; T_max_, time to the maximum grain-filling rate; W_max_, kernel weight at the maximum grain-filling rate; V_mean_, mean grain-filling rate; t_3_, effective grain-filling duration; PH, plant height; LAI, leaf area index; and SDM, shoot dry matter accumulation.

As shown in Figure 6, Pearson correlation analysis revealed highly significant positive correlations among *P*_n_, *G*_s_, and *T*_r_ (*p* < 0.001), whereas *C*_i_ was highly significantly negatively correlated with *P*_n_, *G*_s_, and *T*_r_ (*p* < 0.001). Among the grain-filling parameters, T_max_ was strongly positively correlated with t_3_ (*p* < 0.001), while R_max_ was also significantly positively correlated with V_mean_ and W_max_. PH, LAI, and SDM were highly significantly positively correlated with one another (*p* < 0.001), and SSA also showed strong positive correlations with these three variables. Mantel tests further showed that grain yield was highly significantly associated with 100-kernel weight, starch content, photosynthetic traits, and most grain-filling parameters (*p* < 0.01), and significantly associated with PH, LAI, and SDM (*p* < 0.05), whereas its association with SSA was not significant.

## 3. Discussion

### 3.1. Effects of Maturity Group on Photosynthetic Performance and Dry Matter Accumulation Under Film Mulching

Field studies have demonstrated that modifications of soil management and cultivation practices can alter soil environmental conditions and subsequently influence crop growth and yield formation [28,29]. Film mulching can promote photosynthetic carbon assimilation and dry matter accumulation by improving soil hydrothermal conditions and the root-zone environment and by maintaining leaf water status [30]. In the present study, the photosynthetic traits of maize hybrids under film mulching differed markedly among growth stages and maturity groups. The early-maturing group reached relatively high photosynthetic activity at an earlier stage but exhibited a shorter duration of effective photosynthesis after anthesis. The medium–early-maturing group showed better adaptation to local light and thermal conditions and effective accumulated temperature, and therefore maintained relatively high photosynthetic activity at the milk stage (R3). Although the medium-maturing group developed a larger leaf area and had a longer growth period, its photosynthetic advantage during the late growth stages may have been constrained by insufficient effective accumulated temperature. These findings are generally consistent with those of Wu et al. [31], who suggested that maize photosynthetic performance is jointly regulated by improvements in the soil hydrothermal environment and maturity group. Therefore, the appropriate selection of maturity groups may prolong leaf area duration and the effective photosynthetic period after anthesis, improve the utilization efficiency of light and thermal resources, and promote dry matter accumulation [32].

The present study also showed that, after maize entered R3, net photosynthetic rate (*P*_n_), stomatal conductance (*G*_s_), and transpiration rate (*T*_r_) generally decreased across maturity groups, whereas intercellular CO_2_ concentration (*C*_i_) increased accordingly. Correlation analysis further showed that *C*_i_ was significantly negatively correlated with *P*_n_, *G*_s_, and *T*_r_. These results are generally consistent with those of Guo et al. [33], who attributed such changes to accelerated leaf senescence, declining activity of the photosynthetic apparatus, and weakened carboxylation capacity during the late growth stages. During periods of high photosynthetic activity, leaves have a strong capacity to fix and assimilate intercellular CO_2_, thereby maintaining *C*_i_ at a relatively low level. After R3, however, leaf senescence and reductions in photosynthetic enzyme activity weaken CO_2_ assimilation, resulting in decreased *P*_n_ and increased *C*_i._ Therefore, the reduction in photosynthetic capacity during this period may not be caused primarily by stomatal closure but may instead be related to decreased mesophyll carboxylation efficiency, functional deterioration of the photosynthetic apparatus, and increased non-stomatal limitation [34,35].

### 3.2. Effects of Maturity Group on Grain-Filling Dynamics Under Film Mulching

Grain filling is a critical process governing the transport and accumulation of photosynthates in developing kernels, and both grain-filling rate and duration contribute to final kernel weight, grain yield, and quality formation in maize [36]. In the present study, maize maturity groups exhibited distinct grain-filling characteristics under film mulching. Grain filling began earlier in the early-maturing group, and the time to the maximum grain-filling rate occurred sooner. Kernel dry matter accumulated rapidly during the early and middle grain-filling stages, whereas the increase in kernel weight slowed during the late stage, resulting in a relatively short effective grain-filling duration. The medium–early-maturing group exhibited a higher grain-filling rate and maintained a relatively high grain-filling rate during the main grain-filling period, indicating better coordination between grain-filling rate and duration. By contrast, the medium-maturing group had a longer effective grain-filling duration, but its grain-filling rate was relatively lower than that of the medium–early-maturing group. Thus, the longer effective grain-filling duration of the medium-maturing group did not translate into a corresponding advantage in kernel weight accumulation compared with the medium–early-maturing group.

These results indicate that kernel weight formation in maize from different maturity groups is not determined by a single grain-filling parameter but instead results from the combined effects of grain-filling initiation, grain-filling rate, and effective grain-filling duration [37]. Gambín et al. [38] reported that genetic gains in kernel weight over successive breeding eras were mainly associated with prolonged grain-filling duration in long-season hybrids, whereas increased kernel growth rate contributed more strongly to kernel weight improvement in short-season hybrids. The present results are partially consistent with this pattern. The medium-maturing group exhibited a longer effective grain-filling duration; however, this longer duration was not accompanied by higher grain-filling rates or greater kernel weights than those of the medium–early-maturing group. In contrast, the medium–early-maturing group showed higher grain-filling rates, suggesting that grain-filling rate played a more important role in its kernel weight formation.

The long-season materials examined by Gambín et al. may have been able to fully express their genetic potential for a prolonged grain-filling period under the local experimental conditions. In contrast, the thermal resources available in the present experimental region may not have fully supported rapid assimilate accumulation during the late grain-filling stage of the medium-maturing group. Although film mulching improved soil temperature and moisture conditions during the early and middle growth stages, its warming effect gradually weakened as maize development progressed and may therefore have been insufficient to fully compensate for the decline in air temperature during the late growth stages [39]. Insufficient effective accumulated temperature may reduce leaf photosynthetic activity and assimilate supply during the late growth stages, thereby limiting kernel weight gain. Consequently, although the medium-maturing group maintained a longer effective grain-filling duration, this temporal advantage was not fully converted into higher grain-filling rates or greater kernel weights than those of the medium–early-maturing group [40]. Although grain number per ear is an important component of yield formation, it does not directly determine final grain yield because yield is also strongly influenced by kernel filling capacity. In the present study, the medium–early-maturing group produced more grains per ear, providing a larger sink capacity for yield formation. However, its yield advantage was mainly attributed to the coordination between higher grain number and superior grain-filling performance. In contrast, a greater grain number without sufficient assimilate accumulation and kernel filling capacity may not be fully converted into higher grain yield.

### 3.3. Effects of Maturity Group on Starch Synthase Activity and Starch Accumulation Under Film Mulching

Differences in kernel starch accumulation among maize maturity groups were closely associated with starch synthase activity and the duration for which high enzyme activity was maintained [41]. In the present study, the early-maturing group entered the rapid starch-synthesis stage earlier under film mulching. However, starch synthase activity in this group declined more rapidly during the late grain-filling stage, thereby shortening the period of sustained starch accumulation. In contrast, the medium–early- and medium-maturing groups maintained relatively high starch synthase activity during the middle and late grain-filling stages and consequently exhibited a stronger capacity for sustained starch synthesis. These findings indicate that both the peak level of starch synthase activity and the duration of high activity contribute to starch accumulation and kernel weight formation during the late grain-filling stage [23].

This result is generally consistent with the findings of Yu et al. [42], who reported that differences in starch accumulation capacity among kernels depend not only on the peak activity of starch-synthesizing enzymes but also on the duration for which relatively high enzyme activity is maintained. A stronger capacity to maintain enzyme activity during the middle and late grain-filling stages is conducive to the continued accumulation of starch and kernel dry matter. In addition, starch content and starch synthase activity showed generally consistent responses among maturity groups in the present study, with maturity groups exhibiting higher enzyme activity usually also showing higher starch content. This relationship may be explained by the enhancement of kernel sink capacity for sucrose utilization through increased activities of starch-synthesizing enzymes, thereby promoting the conversion and deposition of sucrose into starch [43]. Han et al. [44] similarly reported that enhanced activities of enzymes involved in starch synthesis increased the starch-synthesis capacity of maize kernels and promoted starch accumulation, whereas inhibition of these enzymes reduced starch deposition.

## 4. Materials and Methods

### 4.1. Experimental Site and Plant Materials

**Figure 7:**
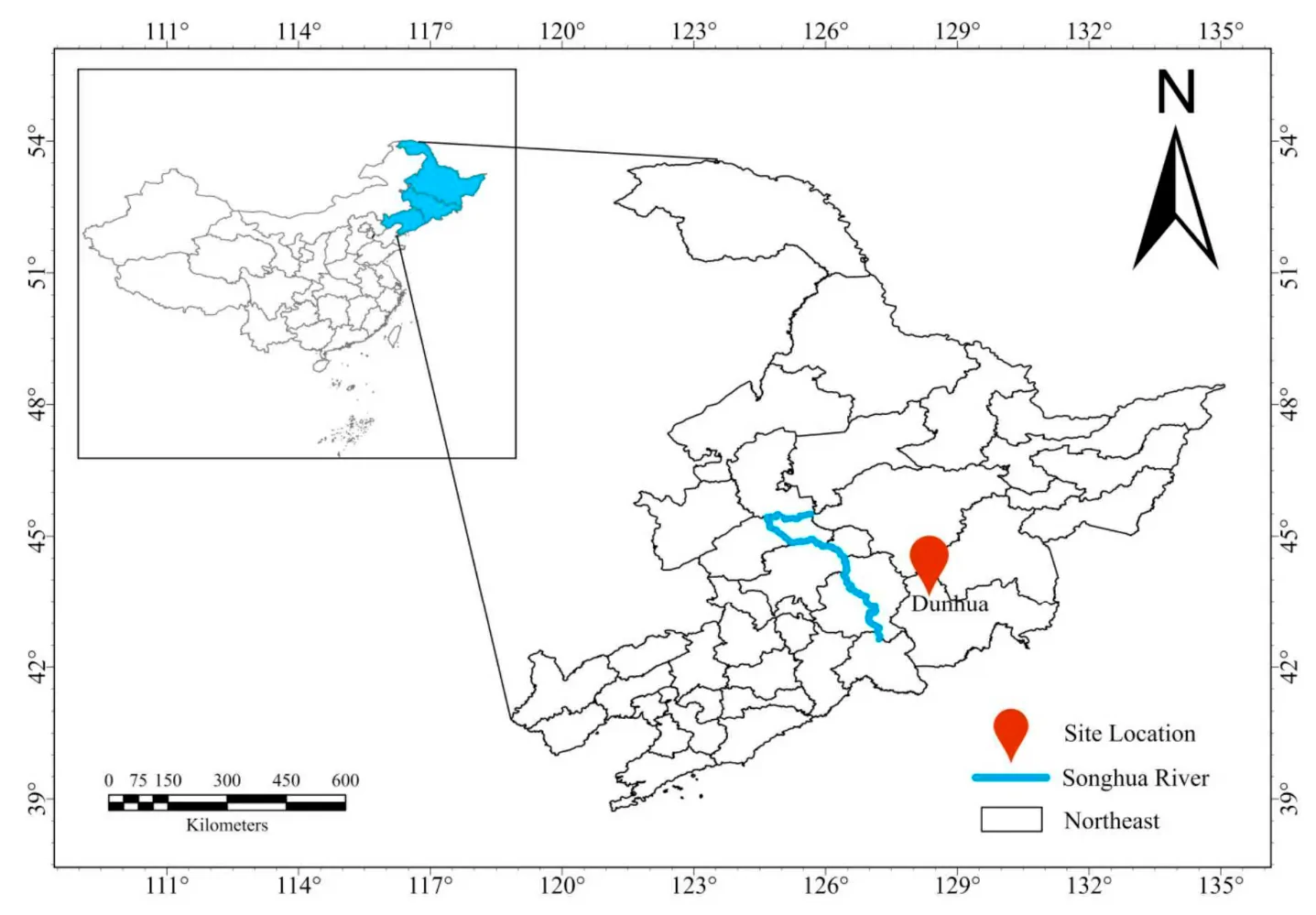
Schematic diagram of the experimental site.

**Figure 8:**
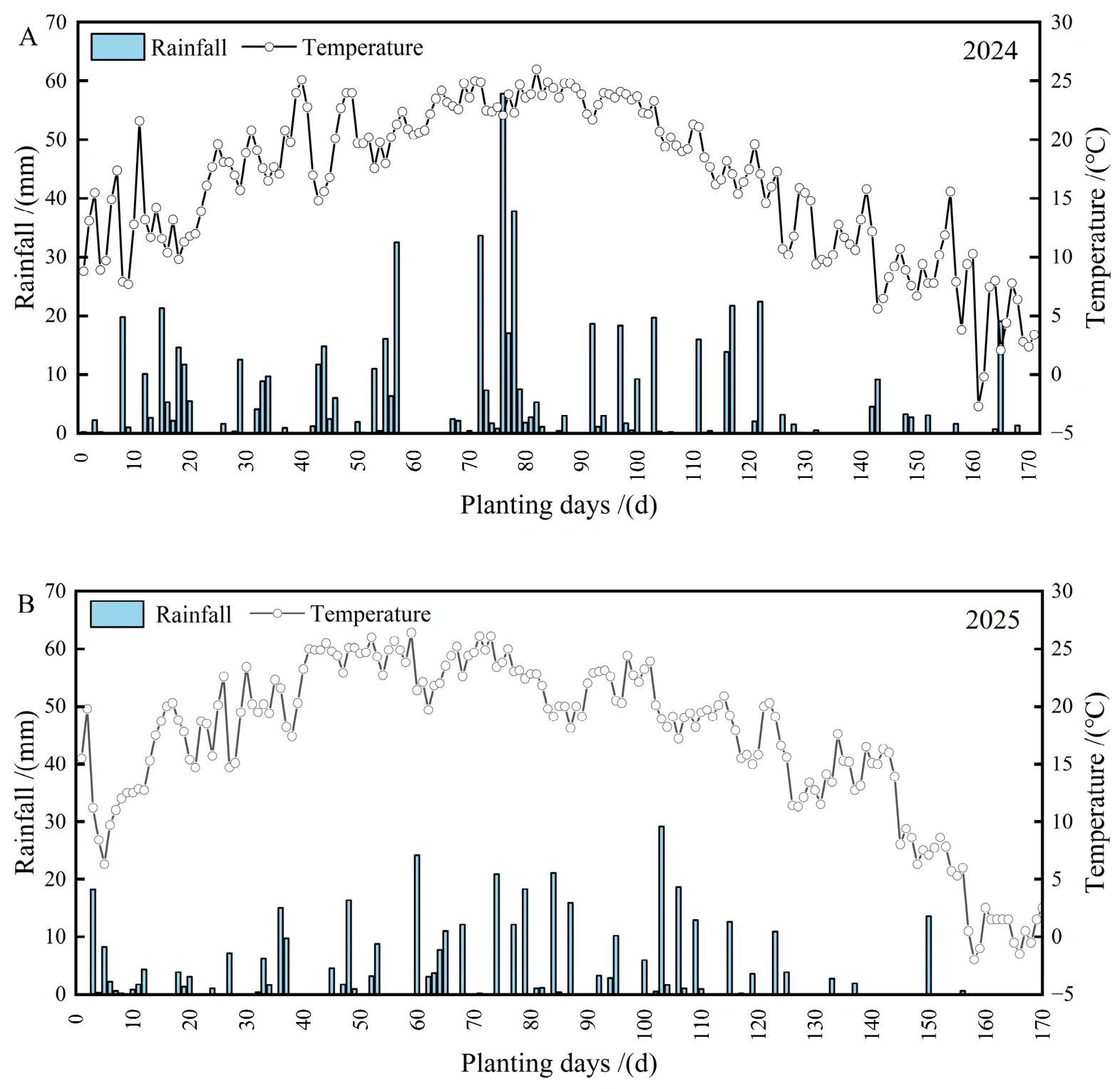
Daily mean air temperature and daily precipitation during the maize growing seasons in 2024 and 2025. (**A**) Daily mean air temperature and precipitation in 2024; (**B**) daily mean air temperature and precipitation in 2025.

The field experiment was conducted from 2024 to 2025 in Shuangshanzi Village, Shaheyan Town, Dunhua City, Yanbian Korean Autonomous Prefecture, Jilin Province, China (128.35° E, 43.44° N; Figure 7). The experimental area has a temperate continental climate, with an annual effective accumulated temperature of 2300–2400 °C·d. The daily mean air temperature and precipitation during the maize growing seasons in 2024 and 2025 are shown in Figure 8. The soil at the experimental site was classified as Albic soil, and the preceding crop was maize. Before the experiment, the basic physicochemical properties of the 0–20 cm soil layer were as follows: soil bulk density, 1.25 g·cm^−3^; soil organic matter, 48.69 g·kg^−1^; total nitrogen, 1.95 g·kg^−1^; total phosphorus, 0.95 g·kg^−1^; total potassium, 2.61 g·kg^−1^; available phosphorus, 28.90 mg·kg^−1^; available potassium, 157.64 mg·kg^−1^; alkali-hydrolyzable nitrogen, 267.18 mg·kg^−1^; and pH, 5.26. Six maize hybrids representing three maturity groups—early-maturing (E), medium–early-maturing (ME), and medium-maturing (M)—were used as the experimental materials, with two hybrids included in each maturity group. The detailed characteristics of the six hybrids are presented in Table 6. All treatments were covered with white PPC biodegradable mulch film manufactured by Jilin Difu Fertilizer Technology Co., Ltd. (Changchun, Jilin Province, China). The mulch film was 90 cm wide and 0.008 mm thick.

### 4.2. Experimental Design

Before sowing, the experimental field was uniformly rotary-tilled and leveled. Film mulching, fertilization, and sowing were completed simultaneously using an integrated agricultural machine. Maize was sown on 5 May 2024 and 7 May 2025 and harvested on 29 October 2024 and 28 October 2025, respectively. The experiment was arranged in a randomized complete block design with six maize hybrids representing three maturity groups and three replicates. Each replicate was considered as a block, and each plot was used as the experimental unit for sampling and statistical analysis. Each plot covered an area of 72 m^2^ (2.4 m × 30 m). A film-mulched wide-ridge, double-row planting pattern was adopted, with two wide ridges in each plot. The spacing between the two rows within each ridge was 0.4 m, and the within-row plant spacing was 0.2 m, resulting in a uniform planting density of 83,333 plants·ha^−1^. A Liuguo controlled-release compound fertilizer formulated for maize was applied as basal fertilizer at a rate of 900 kg·ha^−1^, with an N–P_2_O_5_–K_2_O ratio of 26–11–12. All other field management practices were conducted according to conventional local maize production practices.

### 4.3. Sampling and Measurements

#### 4.3.1. Soil Temperature and Moisture Content

Beginning after maize sowing, soil temperature and moisture content were measured at 10-day intervals at 10:00, 15:00, and 20:00 using an integrated soil temperature–moisture meter. At each sampling time, measurements were taken at soil depths of 10 and 20 cm at fixed locations beneath the mulch and at the uncovered position within each plot. For each sampling date, the mean of the three measurements taken at 10:00, 15:00, and 20:00 was used for statistical analysis and graphical presentation [45].

#### 4.3.2. Leaf Photosynthetic Traits

Leaf photosynthetic traits were measured at the eight-leaf stage (V8), twelve-leaf stage (V12), and milk stage (R3) on clear, cloudless mornings using an LI-6800 portable photosynthesis and fluorescence system (LI-COR, Lincoln, NE, USA). The third fully expanded leaf from the top of the plant was selected for measurements at V8 and V12, whereas the ear leaf was selected at R3. Three consecutive maize plants with uniform growth were selected from each plot. Measurements were conducted at a photosynthetic photon flux density of 1500 μmol·m^−2^·s^−1^. The net photosynthetic rate (*P*_n_), stomatal conductance (*G*_s_), intercellular CO_2_ concentration (*C*_i_), and transpiration rate (*T*_r_) were recorded [46]. The three plants within each plot were considered subsamples, and the mean value was calculated to represent the plot-level value for statistical analysis.

#### 4.3.3. Grain-Filling Characteristics

Kernel samples were collected beginning 10 days after anthesis and subsequently at 10-day intervals, for a total of six sampling dates. At each sampling date, three adjacent plants with uniform growth were selected from a representative location in each plot, and their ears were harvested. A total of 100 kernels were collected from the middle portion of the sampled ears, placed in labeled sample bags, heated at 108 °C for 1 h, and then oven-dried at 80 °C to a constant weight. The 100-kernel weight was determined using an electronic balance with a precision of 0.01 g. Grain-filling rate was calculated as the difference in kernel dry weight between two consecutive sampling dates divided by the 10-day interval, and the resulting rates were assigned to the midpoint of each interval (15, 25, 35, 45, and 55 days after anthesis). The maize grain-filling process was fitted using a Logistic growth model, with days after anthesis (t) as the independent variable and kernel weight (W) as the dependent variable [47]. The grain-filling parameters were calculated as follows:

Maize grain filling equation:

(1) $$W=A/\left(1+Be^{-Ct}\right)$$

Maximum filling rate:

(2) $$R_{\max}=\left(C\times W_{\max}\right)\left(1-W_{\max}/A\right)$$

Time at maximum filling rate:

(3) $$T_{\max}=\ln B/C$$

Growth at maximum filling rate:

(4) $$W_{\max}=A/2$$

Average filling rate:

(5) $$V_{\mathrm{mean}}=W_{3}/t_{3}$$

Start date of peak filling:

(6) $$t_{1}=(\ln B-1.317)/C$$

End date of peak filling:

(7) $$t_{2}=(\ln B+1.317)/C$$

Effective filling time:

(8) $$t_{3}=(\ln B+4.59512)/C$$

Duration of slow filling phase:

(9) $$T_{1}=t_{1}$$

Duration of fast filling phase:

(10) $$T_{2}=t_{2}-t_{1}$$

Duration of slow increment phase:

(11) $$T_{3}=t_{3}-t_{2}$$

Grain weight increment in slow filling:

(12) $$w_{1}=W_{1}$$

Grain weight increment in fast filling:

(13) $$w_{2}=W_{2}-W_{1}$$

Grain weight increment in slow increment phase:

(14) $$w_{3}=W_{3}-W_{2}$$

Average filling rate in slow filling phase:

(15) $$v_{1}=w_{1}/T_{1}$$

Average filling rate in fast filling phase:

(16) $$v_{2}=w_{2}/T_{2}$$

Average filling rate in slow increment phase:

(17) $$v_{3}=w_{3}/T_{3}$$

Note: A: theoretical maximum 100-kernel weight of maize; B, C: shape parameters.

#### 4.3.4. Kernel Starch Synthase Activity and Starch Content

In 2024 and 2025, kernel samples were collected at the silking stage (R1), blister stage (R2), milk stage (R3), dough stage (R4), and dent stage (R5). Three maize plants with uniform growth were selected from each plot, and kernels were collected from the middle portion of each ear. The samples were immediately frozen in liquid nitrogen and stored at −80 °C until analysis.

Starch synthase activity (SSA) was determined using a Plant Starch Synthase (SS) ELISA Kit (catalogue no. MM-35926O2, Jiangsu Meimian Industrial Co., Ltd., Yancheng, China) according to the manufacturer’s instructions. The assay was based on a double-antibody sandwich enzyme-linked immunosorbent assay (ELISA) method. Briefly, starch synthase in the samples was specifically captured by immobilized antibodies and subsequently detected using HRP-labeled antibodies. After substrate incubation and color development, the absorbance was measured at 450 nm, and SSA was calculated according to the standard curve. Each sample was analyzed with three technical replicates, and the mean value was used for subsequent statistical analysis. Starch content (S) was determined using a Starch Content Assay Kit (Anthrone Colorimetric Method, catalogue no. ADS-W-DF001-50, Jiangsu Aidisheng Biological Technology Co., Ltd., Yancheng, Jiangsu, China) according to the manufacturer’s instructions. Briefly, kernel samples were homogenized and extracted following the kit protocol. After extraction, starch was hydrolyzed into glucose under acidic conditions, and the glucose content was quantified using the anthrone colorimetric method. The absorbance was measured at 620 nm using a microplate reader, and S was calculated based on the standard curve. Each sample was analyzed with three technical replicates, and the mean value was used for subsequent statistical analysis. The SSA and S were expressed on a fresh-weight basis as U g^−1^ FW and %, respectively [48].

#### 4.3.5. Agronomic Traits

At V8, V12, and R3, three maize plants with uniform growth were selected from each plot. Plant height and the length and maximum width of each fully expanded leaf were measured using a ruler. Leaf area per plant was calculated as the sum of the areas of all fully expanded leaves according to the following equation: leaf area = leaf length × maximum leaf width × 0.75. After measurement, the sampled plants were separated into individual organs and placed in labeled paper bags. The samples were heated at 105 °C for 30 min and subsequently oven-dried at 80 °C to a constant weight to determine dry matter accumulation per plant [49]. The three plants sampled from each plot were treated as subsamples, and their average values were used as the plot-level observations for subsequent statistical analyses.

#### 4.3.6. Grain Yield and Yield Components

At the physiological maturity stage (R6), three previously unsampled areas were selected from each plot for yield determination, with each harvest area covering 20 m^2^. All ears within each harvest area were collected and weighed. Ten representative ears of similar size were selected for yield-component measurements, including 100-kernel weight and kernel number per ear. Grain yield was adjusted to a standard moisture content of 14% [50].

### 4.4. Data Processing and Analysis

Experimental data were organized using Microsoft Excel for Microsoft 365 (Version 2606; Microsoft Corporation, Redmond, WA, USA) and statistically analyzed using IBM SPSS Statistics 20.0 (IBM Corp., Armonk, NY, USA). The plot was considered as the experimental unit, with three field replicates (*n* = 3). Measurements from multiple plants within each plot were treated as subsamples, and their mean values were used as plot-level observations for statistical analysis. For descriptive comparisons among maturity groups, the mean values of the two hybrids within each maturity group were calculated (E: E1 and E2; ME: ME1 and ME2; M: M1 and M2). These maturity-group means were used to calculate percentage differences and describe variation among maturity groups. Statistical comparisons were conducted among early-maturing, medium–early-maturing, and medium-maturing groups using one-way ANOVA followed by the least significant difference (LSD) test at *p* < 0.05. Two-way or three-way ANOVA was performed to evaluate the main effects and interactions among year, maturity group, growth stage, and other experimental factors where applicable. Mantel tests were conducted to assess the relationships between grain yield and agronomic, physiological, and grain-filling traits. Pairwise distance matrices were constructed using Euclidean distance, and Mantel tests were performed using the vegan package in R (version 4.6.1) with 999 permutations. Grain-filling parameters were obtained by fitting the Logistic equation using CurveExpert 1.4. Figures and tables were prepared using OriginPro 2024 (Version 10.1), Microsoft Excel for Microsoft 365, and R 4.6.1, whereas the study-area map was generated using ArcGIS Pro 3.6.

## 5. Conclusions

The two-year field experiment conducted in 2024 and 2025 showed that, under uniform film mulching, maize hybrids from different maturity groups differed markedly in photosynthetic performance, dry matter accumulation, grain filling, and starch synthesis. The early-maturing hybrids progressed rapidly through their growth cycle and initiated starch accumulation earlier; however, their shorter effective photosynthetic period after anthesis and shorter effective grain-filling duration limited kernel weight accumulation during the late grain-filling stage. Although the medium-maturing hybrids developed a larger leaf area and maintained a longer effective grain-filling duration, their relatively lower grain-filling rate and the possible limitation of late-season thermal conditions may have constrained the conversion of biomass accumulation and enzyme activity advantages into greater kernel weight and grain yield. In contrast, the medium–early-maturing hybrids maintained relatively high photosynthetic capacity at V12 and R3 and accumulated more shoot dry matter at R3. Their Rmax, Wmax, and Vmean were 10.0%, 12.1%, and 7.2% higher, respectively, than those of the early-maturing hybrids, and 5.4%, 2.8%, and 9.0% higher, respectively, than those of the medium-maturing hybrids. They also maintained stronger starch synthesis capacity during the middle and late grain-filling stages. Averaged across the two years, their grain yield was 20.0% and 9.1% higher than that of the early- and medium-maturing hybrids, respectively. Overall, the medium–early-maturing hybrids achieved better coordination among canopy photosynthesis, dry matter accumulation, grain filling, and starch synthesis, resulting in superior yield performance under biodegradable film mulching. Their growth and developmental characteristics showed better adaptation to the thermal conditions of cool regions in Northeast China, where the annual effective accumulated temperature is approximately 2300–2400 °C·d. Therefore, the medium–early maturity group may represent a suitable choice for balancing maturity stability and high yield potential under biodegradable film mulching in this region.

## Figures and Tables

**Table 1:** Significance of the main effects and interactions of year, maturity group, and growth stage on net photosynthetic rate (Pn), stomatal conductance (Gs), transpiration rate (Tr), and intercellular CO_2_ concentration (Ci).

Source	*P*_n_	*G*_s_	*T*_r_	*C*_i_
Year (Y)	***	***	***	**
Maturity group (M)	***	***	***	***
Growth stage (G)	***	***	***	***
Year (Y) × Maturity group (M)	ns	ns	ns	ns
Year (Y) × Growth stage (G)	***	ns	*	**
Maturity group (M) × Growth stage (G)	**	**	**	**
Year (Y) × Maturity group (M) × Growth stage (G)	ns	ns	ns	ns

Note: *, **, and *** indicate significant effects at *p* < 0.05, *p* < 0.01, and *p* < 0.001, respectively; ns indicates a nonsignificant effect.

**Table 2:** Grain-filling parameters of maize maturity groups under film mulching.

Year	Hybrid Code	Logistic Model	R_max_ (g·d^−1^)	T_max_ (d)	W_max_ (g)	V_mean_ (g·d^−1^)	t_3_ (d)
2024	E1	W = 30.65910/(1 + 12.07239e^−0.10157t^)	0.78 e	24.52 e	15.33 e	0.44 cd	69.76 d
	E2	W = 29.86891/(1 + 12.24418e^−0.10281t^)	0.77 e	24.37 f	14.93 f	0.43 d	69.06 e
	ME1	W = 33.55373/(1 + 16.29530e^−0.10714t^)	0.90 a	26.05 d	16.78 a	0.49 a	68.94 f
	ME2	W = 32.68504/(1 + 15.55447e^−0.10471t^)	0.86 b	26.21 c	16.34 c	0.47 b	70.1 c
	M1	W = 33.25607/(1 + 18.30744e^−0.10044t^)	0.84 c	28.94 b	16.63 b	0.45 c	74.69 b
	M2	W = 32.09236/(1 + 18.98534e^−0.10021t^)	0.80 d	29.37 a	16.05 d	0.43 d	75.23 a
2025	E1	W = 29.42718/(1 + 11.83806e^−0.11303t^)	0.83 cd	21.86 e	14.71 e	0.47 bc	62.52 e
	E2	W = 28.64136/(1 + 11.76860e^−0.11411t^)	0.82 d	21.61 f	14.32 f	0.46 cd	61.88 f
	ME1	W = 33.87622/(1 + 13.30146e^−0.10483t^)	0.89 a	24.69 c	16.94 a	0.49 a	68.52 c
	ME2	W = 32.79492/(1 + 13.31351e^−0.10569t^)	0.87 b	24.49 d	16.4 b	0.48 ab	67.97 d
	M1	W = 32.36355/(1 + 20.50251e^−0.10682t^)	0.86 b	28.42 b	16.18 c	0.45 de	71.66 a
	M2	W = 31.53439/(1 + 20.81802e^−0.10666t^)	0.84 c	28.46 a	15.77 d	0.44 e	71.54 b
Year (Y)			**	**	ns	**	**
Maturity group (M)			**	**	**	**	**
Year × Maturity group (Y × M)			**	**	ns	ns	**

Note: *R*_max_, maximum grain-filling rate; *T*_max_, time to the maximum grain-filling rate; *W*_max_, kernel weight at the maximum grain-filling rate; *V*_mean_, mean grain-filling rate; and *t*_3_, effective grain-filling duration. Different lowercase letters within the same column indicate significant differences among the six individual maize hybrids within the same year at *p* < 0.05. ** indicate significant effects at *p* < 0.01, respectively; ns indicates a nonsignificant effect.

**Table 3:** Stage-specific grain-filling parameters of maize maturity groups under film mulching.

Year	Filling Phase	Parameters	E1	E2	ME1	ME2	M1	M2
2024	Early stage	T_1_ (d)	11.56 e	11.56 e	13.76 c	13.63 d	15.83 b	16.23 a
		v_1_ (g·d^−1^ 100-kernel^−1^)	0.56 a	0.55 a	0.52 b	0.51 b	0.44 c	0.42 d
		w_1_ (g·100-kernel^−1^)	6.48 e	6.31 f	7.09 a	6.91 c	7.03 b	6.78 d
	Middle stage	T_2_ (d)	24.16 c	23.87 d	22.91 f	23.44 e	24.43 b	24.49 a
		v_2_ (g·d^−1^ 100-kernel^−1^)	0.69 e	0.68 e	0.80 a	0.76 b	0.74 c	0.72 d
		w_2_ (g·100-kernel^−1^)	16.73 e	16.30 f	18.31 a	17.84 c	18.15 b	17.52 d
	Late stage	T_3_ (d)	34.05 c	33.64 d	32.28 e	33.03 f	34.43 b	34.51 a
		v_3_ (g·d^−1^·100-kernel^−1^)	0.22 c	0.22 c	0.25 a	0.24 a	0.23 bc	0.23 bc
		w_3_ (g·100-kernel^−1^)	7.45 e	7.25 f	8.15 a	7.94 c	8.08 a	7.79 d
2025	Early stage	T_1_ (d)	10.21 e	10.06 f	12.12 c	12.03 d	16.03 b	16.11 a
		v_1_ (g·d^−1^ 100-kernel^−1^)	0.61 a	0.60 ab	0.59 bc	0.58 c	0.43 d	0.41 d
		w_1_ (g·100-kernel^−1^)	6.22 e	6.05 f	7.16 a	6.93 b	6.84 c	6.66 d
	Middle stage	T_2_ (d)	21.71 e	21.51 f	23.41 a	23.22 b	23.09 c	23.01 d
		v_2_ (g·d^−1^ 100-kernel^−1^)	0.74 cd	0.73 d	0.79 a	0.77 b	0.77 b	0.75 c
		w_2_ (g·100-kernel^−1^)	16.06 e	15.63 f	18.49 a	17.90 b	17.66 c	17.21 d
	Late stage	T_3_ (d)	30.59 e	30.31 f	32.99 a	32.72 b	32.54 c	32.42 d
		v_3_ (g·d^−1^·100-kernel^−1^)	0.23 b	0.23 b	0.25 a	0.24 ab	0.24 ab	0.24 ab
		w_3_ (g·100-kernel^−1^)	7.15 e	6.96 f	8.23 a	7.96 b	7.86 c	7.66 d

Note: T_1_, duration of the gradual-increase stage; T_2_, duration of the rapid-increase stage; T_3_, duration of the slow-increase stage; v_1_, mean grain-filling rate during the gradual-increase stage; v_2_, mean grain-filling rate during the rapid-increase stage; v_3_, mean grain-filling rate during the slow-increase stage; w_1_, kernel weight increment during the gradual-increase stage; w_2_, kernel weight increment during the rapid-increase stage; and w_3_, kernel weight increment during the slow-increase stage. Different lowercase letters within the same column indicate significant differences among maturity groups at *p* < 0.05.

**Table 4:** Significance of the main effects and interactions of year, maturity group, and growth stage on plant height (PH), leaf area index (LAI), and shoot dry matter accumulation (SDM).

Source	PH	LAI	SDM
Year (Y)	ns	**	**
Maturity group (M)	**	**	**
Growth stage (G)	**	**	**
Year (Y) × Maturity group (M)	ns	ns	ns
Year (Y) × Growth stage (G)	ns	**	**
Maturity group (M) × Growth stage (G)	ns	ns	**
Year (Y) × Maturity group (M) × Growth stage (G)	ns	*	ns

Note: * and ** indicate significant effects at *p* < 0.05 and *p* < 0.01, respectively; ns indicates a nonsignificant effect.

**Table 5:** Grain yield and yield components of maize maturity groups under film mulching.

Year	Hybrid Code	Number of Grains Per Ear	100-Grain Mass/(g)	Yield/(kg·hm^−2^)
2024	E1	555.86 ± 28.44 c	29.69 ± 1.63 a	10,899.25 ± 568.76 c
	E2	574.08 ± 24.67 bc	30.52 ± 1.26 a	11,558.44 ± 752.25 bc
	ME1	618.24 ± 24.97 ab	31.28 ± 1.52 a	13,070.93 ± 238.78 a
	ME2	637.01 ± 31.12 a	31.80 ± 0.64 a	13,211.82 ± 397.23 a
	M1	575.18 ± 19.60 bc	33.85 ± 0.06 a	11,998.86 ± 185.41 b
	M2	602.78 ± 28.41 abc	34.36 ± 2.06 a	12,067.44 ± 584.41 b
2025	E1	560.65 ± 26.55 b	30.05 ± 1.62 c	10,421.38 ± 324.85 d
	E2	561.38 ± 27.63 b	30.67 ± 0.55 bc	11,311.04 ± 434.56 c
	ME1	633.51 ± 27.25 a	31.71 ± 0.86 bc	13,194.31 ± 124.54 a
	ME2	650.26 ± 24.98 a	32.14 ± 3.30 bc	13,537.86 ± 358.92 a
	M1	567.64 ± 34.94 b	33.53 ± 0.44 ab	12,118.75 ± 544.55 b
	M2	604.62 ± 16.19 ab	35.89 ± 1.81 a	12,410.89 ± 209.89 b
Year (Y)		ns	ns	ns
Maturity group (M)		**	**	**
Year × Maturity group (Y × M)		ns	ns	ns

Note: Different lowercase letters within the same column indicate significant differences among maturity groups within the same year at *p* < 0.05. ** indicate significant effects at *p* < 0.01, respectively; ns indicates a nonsignificant effect.

**Table 6:** Experimental materials and characteristics of the maize hybrids.

Hybrid Code	Hybrid	Maturity Group	Variety Approval No.	Growth Duration (d)	Seed Supplier
E1	Dika 226	Early-maturing	Guoshenyu 20242034	119.7	China National Seed Group International Seed Co., Ltd., Beijing, China
E2	Jidan 494	Early-maturing	Jishenyu 20253003	118	Jilin Academy of Agricultural Sciences, Changchun, China
ME1	Jidan 407	Medium–early-maturing	Mengshenyu 2018013	124	Jilin Academy of Agricultural Sciences, Changchun, China
ME2	Jidan 626	Medium–early-maturing	Jishenyu 20210050	123	Jilin Academy of Agricultural Sciences, Changchun, China
M1	Jidan 436	Medium-maturing	Guoshenyu 20210019	130	Jilin Academy of Agricultural Sciences, Changchun, China
M2	Jidan 418	Medium-maturing	Jishenyu 20210057	128	Jilin Academy of Agricultural Sciences, Changchun, China

## Data Availability

All relevant data are contained within the manuscript.
